# USING ADJUSTED MULTIPLE CORRESPONDENCE ANALYSIS TO EXPLORE LATENT VARIABLES FOR THE DEMPOL-Q

**DOI:** 10.1093/geroni/igac059.701

**Published:** 2022-12-20

**Authors:** Anna-Louisa Hoffmann, Johannes-Michael Bergmann, Bernhard Holle, Martina Roes, Rebecca Palm

**Affiliations:** German Center for Neurodegenerative Diseases (DZNE), Witten, Nordrhein-Westfalen, Germany; German Center for Neurodegenerative Diseases (DZNE), Göttingen, Nordrhein-Westfalen, Germany; German Center For Neurodegenerative Diseases (DZNE), Witten, Nordrhein-Westfalen, Germany; German Center for Neurodegenerative Diseases (DZNE), Witten, Nordrhein-Westfalen, Germany; University of Witten/Herdecke, Witten, Nordrhein-Westfalen, Germany

## Abstract

Nursing home administrators are responsible to operationalize dementia-specific person-centred care into their care practice, e.g. by internal policies. To assess the degree of existence of internal policies on person-centred care for dementia-specific care in German nursing homes, we developed the Dementia Policy Questionnaire (DemPol-Q). After pretesting, the instrument consists of 19 dichotomous items. We aimed to explore the construct validity of the DemPol-Q. We used a secondary data set of a national survey with a representative sample of 134 nursing homes. For data analysis we conducted an Adjusted Multiple Correspondence Analysis. Results show that nine items of the DemPol-Q were significantly assigned to two latent variables. Since the items per latent variable vary in their content, they cannot be explicit denominated with a specific sub-dimension of dementia-specific person-centred care. Nonetheless, Adjusted Multiple Correspondence Analysis is an uncommon but appropriate exploratory data reduction procedure for nursing science.

